# Integrating the patient voice with clinician reports to identify a hepatocellular carcinoma-specific subset of treatment-related symptomatic adverse events

**DOI:** 10.1186/s41687-018-0063-4

**Published:** 2018-08-22

**Authors:** Rebecca M. Speck, William R. Lenderking, James W. Shaw

**Affiliations:** 1 Evidera, Bethesda, MD USA; 2 Bristol-Myers Squibb, 3401 Princeton Pike, Lawrenceville, NJ 08648 USA

**Keywords:** Symptomatic adverse events, Patient-reported outcomes, Hepatocellular carcinoma, PRO-CTCAE

## Abstract

**Background:**

Incorporating patient reporting of symptomatic adverse events (AEs) is important in evaluating safety and tolerability in cancer clinical trials. The Patient-Reported Outcomes version of the Common Terminology Criteria for Adverse Events (PRO-CTCAE) was developed to assess the frequency, severity, and/or interference of patient-reported symptomatic AEs. The objective of this study was to identify, based on oncologist and patient interviews, a relevant subset of symptomatic AEs from the PRO-CTCAE that can be used to optimize patient reporting of symptomatic AEs in hepatocellular carcinoma (HCC) clinical trials.

**Methods:**

Qualitative and quantitative data on HCC diagnosis, treatment, symptoms, and side effects were collected from patients. Using a numerical rating scale, medical oncologists specializing in HCC rated the importance of (1) 34 symptomatic AEs (Grade ≥2) identified in past sponsor HCC clinical trials, (2) each PRO-CTCAE symptomatic AE item to the patient, and (3) each PRO-CTCAE symptomatic AE item with respect to patient safety or tolerability. Patients completed the PRO-CTCAE items and were debriefed on the importance of each PRO-CTCAE symptomatic AE to them.

**Results:**

Five medical oncologists from the United States, Spain, Taiwan, Korea, and Hong Kong with 14 to 30 years of experience, and 17 patients with HCC and Child-Pugh class A or B cirrhosis status completed interviews. Medical oncologists rated the following symptomatic AEs from prior trials as being highly important to patients (mean rating of ≥7 on a scale from 0 to 10): hand-foot syndrome, diarrhea, fatigue, decreased appetite, rash, vomiting, and weight loss. PRO-CTCAE symptomatic AEs rated by medical oncologists as being highly important to patients included diarrhea, vomiting, shivering or shaking chills, hand-foot syndrome, rash, fatigue, difficulty swallowing, decreased appetite, and loss of control of bowel movements. Patients rated the following PRO-CTCAE symptomatic AEs as being highly important: loss of appetite/lack of interest in food, pain/tenderness at injection/insertion site, fatigue/lack of energy/tiredness, nausea, and hair loss.

**Conclusions:**

This study identified a preliminary list of clinically relevant symptomatic AEs from interviews with both medical oncologists and patients that can be used to support assessments of treatment safety and tolerability in HCC clinical trials.

## Background

Cancer treatments are often associated with symptomatic adverse events (AEs), and a more robust assessment of these events has important clinical and scientific value [1]. In cancer clinical trials, clinicians and study researchers commonly use the Common Terminology Criteria for Adverse Events (CTCAE) to grade and report AEs; it is composed of approximately 790 AEs graded on a severity scale (0 to 5) [2]. With the CTCAE, patient signs and symptoms are interpreted and reported by clinicians [3]. Given the subjective nature of many CTCAE criteria, differentiation of Grade 2 and 3 symptomatic AEs can be ambiguous [4], and it has been demonstrated that the reliability of CTCAE grading among providers is not robust. For example, examination of interobserver agreement of CTCAE criteria showed that independent providers agreed on the grading of fewer than half of all patients (45.9%) [5]. Furthermore, clinicians often underreport symptomatic events [6–8].

The Patient-Reported Outcomes version of the CTCAE (PRO-CTCAE) was developed to assess the frequency, severity, and/or interference of symptomatic AEs as directly reported by patients (https://healthcaredelivery.cancer.gov/pro-ctcae/). It measures 78 symptomatic AEs drawn from the CTCAE and was developed to complement the CTCAE data from clinical trials [3, 9, 10]. Akin to the CTCAE, the PRO-CTCAE assesses symptomatic AEs that have relevance across therapies and disease states. In any application of the PRO-CTCAE, it is important to ensure that items assessing all relevant symptomatic AEs are included. However, the exclusion of irrelevant items is also important to minimize respondent burden and measurement noise, and would better allow symptomatic AEs that are important to patients to be emphasized. Although several recommendations have been put forward for selecting subsets of items from the PRO-CTCAE that are relevant in a specific therapeutic context, including criteria based on the patient perspective, a consensus has not been reached [11, 12].

For many patients with advanced malignancies, there is currently a high unmet need for safe and effective therapies, and many currently available treatments have problematic side effects. Patients with hepatocellular carcinoma (HCC) are often diagnosed at advanced stages and have poor outcomes [13]. Sorafenib is approved for first-line treatment of patients with advanced HCC; regorafenib and nivolumab are approved as second-line treatments (nivolumab in the United States only). All 3 treatments have notable side effects [14–16]. Sorafenib has been associated with diarrhea, hand-foot skin reactions, and weight loss [14], regorafenib has been associated with hand-foot skin reactions, diarrhea, and fatigue [15], and nivolumab has been associated with diarrhea, pruritus, and rash [16]. Safety assessments in clinical trials could be better focused through patient and clinician identification of key symptomatic AEs with the use of the PRO-CTCAE.

Here we report results from a mixed-methods investigation, in which input from medical oncologists who treat patients with HCC and from patients with HCC was integrated with clinical trial data to identify a subset of symptomatic AEs from the PRO-CTCAE that were clinically relevant to HCC therapies and important to patients enrolled in HCC clinical trials. The approach described in this report enabled comparisons between patient and provider perceptions of symptomatic AEs in HCC, which could be used as a template for future studies.

## Methods

### Study population

This study analyzed cross-sectional, mixed-methods data that were collected from both clinical experts and patients with HCC. HCC clinical experts were 5 international medical oncologists from 5 locations (United States, Spain, Taiwan, Korea, and Hong Kong) with experience ranging from 14 to 30 years. The analysis patient population included 17 male and female English-speaking adult patients from the United States with HCC. Eligible patients had Child-Pugh class A or B disease, had hepatitis B or C virus infection or were uninfected, and had an Eastern Cooperative Oncology Group performance status of 0 or 1. Patients were ineligible if they had known fibrolamellar HCC or mixed cholangiocarcinoma and HCC, active co-infection with both hepatitis B and C viruses, and viral hepatitis other than type B or C. Other exclusion criteria were brain metastases or suspected brain metastases, concomitant second malignancies, uncontrolled or significant cardiac disease, prior liver transplant, known anti–programmed death 1/programmed death ligand 1 treatment, or any other mental or physical condition that in the judgment of study staff would impair the patient’s ability to participate in cognitive interviews or complete study questionnaires. Patients were recruited from 5 third-party contracted clinical sites; the study sponsor was blinded to the name and location of clinical sites, and the sites were blinded to the study sponsor. This study was approved by the Chesapeake Institutional Review Board (protocol 00013983). Patients provided written informed consent.

### Data collection

Data were collected from medical oncologists with semi-structured, one-on-one, qualitative telephone interviews of 45 to 60 min. Medical oncologists were asked open-ended questions about HCC diagnostic pathways, treatment options, symptoms, and common treatment-related symptomatic AEs. Following the interview, they used a numerical rating scale to rate the perceived importance to patients (i.e., how burdensome) of 34 symptomatic AEs, on a scale of 0 (unimportant) to 10 (important). The 34 symptomatic AEs (Table 1) were selected based on safety data from prior phase 3 clinical trials (BRISK-FL (Brivanib Versus Sorafenib as First-line Treatment in Patients With Advanced HCC; NCT00858871), BRISK-PS (Brivanib Plus Best Supportive Care Versus Placebo Plus Best Supportive Care in Patients With Advanced HCC Who Have Failed or Are Intolerant to Sorafenib; NCT00825955), and BRISK-TA (Brivanib Versus Placebo as Adjuvant Therapy to Trans-Arterial Chemo-Embolization in Patients With Unresectable HCC; NCT00908752)). From these trials, symptomatic AEs were selected for review if they had a Grade ≥2.

**Table 1:** List of 34 symptomatic AEs ≥2 from sponsor trials rated by oncologists in one-on-one interviews

Hypotension	Diarrhea	Nausea	Vomiting
Fatigue	Decreased appetite	Anemia	Hypothyroidism
Dyspepsia	Back pain	Muscle weakness	Headache
Dizziness	Dysgeusia	Insomnia	Constipation
Stomatitis	Abdominal pain	Dysphonia	Dyspnea
Cough	Hand-foot syndrome	Rash	Pruritus
Dry skin	Ascites	Dry mouth	Abdominal distention
Asthenia	Mucosal inflammation	Peripheral edema	Pyrexia
Weight loss	Dehydration		

The medical oncologists also used a 0–10 numerical scale to rate the importance of all items within the PRO-CTCAE (v1.0), first with respect to safety or tolerability (i.e., dose limiting), then based on how important (i.e., how burdensome) they perceived the item would be to patients. An average pooled oncologist rating score ≥7 was selected as the cutoff for inclusion based on 70% (i.e., approximately two-thirds) of the total scale range. The team felt that this would optimize the balance between being inclusive and selecting a practical number of items for use within clinical trials.

Data were collected from patients with semi-structured, one-on-one, qualitative telephone interviews of 60 to 90 min. Patients were asked open-ended, concept-elicitation questions about their HCC diagnosis, the process by which they were diagnosed, the HCC symptoms they experienced, the types of treatment received, any treatment side effects, and the impact of symptomatic AEs on their quality of life. Patients also completed the PRO-CTCAE and underwent cognitive debriefing about the importance and relevance of each PRO-CTCAE item in the context of their experience with HCC. Following completion of the interview, patients completed a sociodemographic form, and the clinical sites completed brief case report forms to collect information on HCC treatments received, comorbidities, and current medications.

### Data analysis

Audio responses from patient interviews were transcribed by a professional transcription service. Upon receipt of interview transcripts, all protected health information was deidentified, and a quality review of each transcript was conducted. A coding dictionary was developed to assist with review and analysis of the qualitative data.

A content analysis approach was used to assess qualitative data obtained from the patient interviews using ATLAS.ti software v7.0 (or later) [17], designed for the qualitative analysis of textual, graphical, audio, and video data. ATLAS.ti databases allow researchers to create concepts, or “codes,” that are used to organize and systematically analyze and compare large amounts of qualitative data to identify underlying themes and relationships.

For the concept-elicitation phase of the study, saturation was evaluated by entering ATLAS.ti codes for HCC-related symptoms and treatment-related symptomatic AEs into “saturation grids” that tracked the content of the interviews. Saturation is said to occur when no new information is gained during several sequential interviews [18, 19]. In this study, saturation was defined when no new symptomatic AEs were mentioned in two consecutive interviews. Concepts obtained from patient interviews were compared with the content of the PRO-CTCAE to determine whether there was appropriate content coverage. For the cognitive interview portion of the study, patient responses were coded to examine comprehension, relevance, and ease or difficulty of selecting a response for each item on the PRO-CTCAE.

## Results

**Fig. 1:**
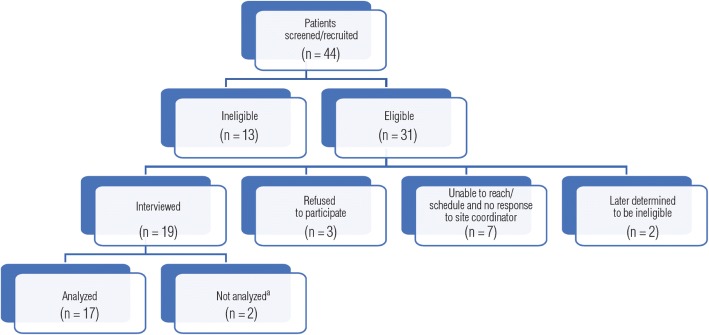
Patient recruitment. ^a^Interview audio files were not transcribed because patient did not self-report having received any treatment for HCC

Figure 1 presents a schematic diagram of patient recruitment. This was a challenging population to recruit and interview. Of 44 patients initially screened, 31 (70.4%) were considered eligible to participate. Of these, 17 (54.8%) were ultimately interviewed and analyzed. Among the 13 patients who were ineligible, 8 were medically ineligible, 1 died before he or she could be interviewed, 1 was psychiatrically ineligible, and 3 were listed as ineligible by the site because they did not want to participate. Of the 31 eligible patients, 3 did not want to participate, 7 could not be reached, 2 were later judged ineligible, and 19 were interviewed; however, 2 interviews were not analyzed due to disclosure of exclusion factors during the interview.

### Medical oncologist assessments

Medical oncologists who were interviewed about treating patients with HCC were asked, “What are the most common symptomatic treatment-related toxicities?” Their summarized responses are presented in Table 2. Four of the 5 medical oncologists discussed symptomatic AEs related specifically to treatment with sorafenib, including skin toxicity (hand-foot syndrome, rash), diarrhea, fatigue, hypertension, and loss of appetite. The symptomatic AEs mentioned that were related to post–transcatheter chemoembolization (TACE) included pain (abdominal pain), fever, and chills.

**Table 2:** Medical oncologist summarized responses

001	Variable based on the type of treatment. Surgery, radiation, chemotherapy, targeted therapies. Overall, the most common toxicity is fatigue or pain.
002	There are 4 main adverse events with sorafenib and a variety of others that are fairly uncommon. The 4 main symptoms are fatigue, skin toxicity (hand-foot syndrome or rash), diarrhea, and arterial hypertension.
003	With sorafenib, rash is the most common. We haven’t really seen cumulative toxicity with immune therapies, though, because patients usually haven’t been on treatment more than 3 months.
004	Post–transcatheter arterial chemoembolization (TACE) syndrome can occur, which includes fever, chills, abdominal pain and would last about 3 to 7 days. Sorafenib has significant skin toxicity—hand-foot syndrome and diarrhea, which can be very severe. Hypertension is another side effect, although it is not symptomatic. Skin toxicity is very typical with sorafenib and is definitely not related to the disease.
005	Patients will have pain, fatigue, and nausea after local-regional therapy. Patients who receive sorafenib experience fatigue, loss of appetite, diarrhea (about 10% have significant diarrhea), skin toxicities, rashes, and some hypertension.

Symptomatic AEs identified from prior clinical trials that oncologists rated as being most important (average score ≥7) to patients included hand-foot syndrome (8.60), diarrhea (7.80), fatigue (7.80), rash (7.40), decreased appetite (7.20), vomiting (7.20), and weight loss (7.00). The PRO-CTCAE symptomatic AEs that medical oncologists rated as being most important to patients were hand-foot syndrome (8.40), diarrhea (8.40), rash (8.00), fatigue (7.80), loss of control of bowel movements (7.60), vomiting (7.40), difficulty swallowing (7.20), shivering/shaking chills (7.20), and decreased appetite (7.00), whereas those rated most important for assessment of patient safety and tolerability were diarrhea (8.40) and shivering/shaking chills (7.40).

### Patient-based assessments

The baseline demographics and clinical characteristics of patients with HCC recruited into the study are shown in Table 3. Patients had a mean age of 69 years (range, 54–84 years), and most (88%) were male. More than half (53%) of the patients self-reported that their general health was “good” or “very good.” Patients had received one or more different HCC treatments, which included chemotherapy (11.8%), TACE (29.4%), external radiation (5.9%), ablation (47.1%), liver resection (23.5%), Y90 radioembolization (35.3%), and sorafenib (29.4%). Nine (52.9%) patients had received one treatment, five (29.4%) had received two, one (5.9%) had received three, and two (11.8%) patients had received four different HCC treatments.

**Table 3:** Patient characteristics

	Total
	(*N* = 17)
Age, years	
Mean (SD)	68.8 (8.5)
Median	68
Min-max	54–84
Sex	
Male	15 (88.2%)
Female	2 (11.8%)
Race	
White	13 (76.4%)
Black or African American	2 (11.8%)
Asian	0
Native Hawaiian or other Pacific Islander	0
American Indian or Alaskan native	0
Other	1 (5.9%)
Not reported	1 (5.9%)
Ethnicity	
Hispanic or Latino	2 (11.8%)
Not Hispanic or Latino	13 (76.4%)
Not reported	2 (11.8%)
Living situation	
Living alone	6 (35.3%)
Living with partner or spouse, family, or friends	9 (52.9%)
Other	1 (5.9%)
Not reported	1 (5.9%)
Employment status	
Employed full time	1 (5.9%)
Employed part time	0
Unemployed	1 (5.9%)
Retired	10 (58.8%)
Disabled	2 (11.8%)
Other	2 (11.8%)
Not reported	1 (5.9%)
Education level^a^	
Secondary/high school	2 (11.8%)
Some college	7 (41.2%)
College degree	4 (23.5%)
Postgraduate degree	2 (11.8%)
Other or Missing	2 (11.8%)
General health (past week)	
Very good	3 (17.7%)
Good	6 (35.3%)
Fair	5 (29.4%)
Poor	2 (11.8%)
Very poor	0
Not reported	1 (5.9)
Child-Pugh class	
A—with no locoregional treatment	3 (17.7%)
A—with locoregional treatment but before sorafenib treatment	4 (23.5%)
A—after sorafenib treatment	3 (17.7%)
B—with any treatment experience	7 (41.2%)
ECOG performance status	
0	8 (47.1%)
1	9 (52.9%)
Comorbidities^a^	
Asthma	2 (11.8%)
Anxiety/depression	5 (29.4%)
Arthritis	3 (17.7%)
COPD (emphysema, chronic bronchitis)	3 (17.7%)
Diabetes	4 (23.5%)
Hypertension (high blood pressure)	10 (58.8%)
Chest pain/abdominal pain	1 (5.9%)
No other health conditions	2 (11.8%)
HCC treatments received^a^	
Chemotherapy	2 (11.8%)
Transarterial chemoembolization	5 (29.4%)
External radiation	1 (5.9%)
Ablation	8 (47.1%)
Liver resection	4 (23.5%)
Y90 radioembolization	6 (35.3%)
Sorafenib	5 (29.4%)
Current medications^a^	
Antibiotics	2 (11.8%)
Nutritional support	2 (11.8%)
Pain medications/corticosteroids/anti-inflammatory agents	5 (29.4%)
Drugs for gastrointestinal-related disorders	6 (35.3%)
Drugs for anemia	1 (5.9%)
None	2 (11.8%)

COPD, chronic obstructive pulmonary disease; ECOG, Eastern Cooperative Oncology Group; HCC, hepatocellular carcinoma

^a^Not mutually exclusive

During the qualitative interviews, a total of 33 symptomatic AEs were reported spontaneously by patients. Symptomatic AEs reported by ≥4 (i.e., ~25%) of the patients were fatigue/lack of energy/tiredness (*n* = 10), loss of appetite/lack of interest in food (*n* = 6), nausea (*n* = 6), pain/tenderness at injection/insertion site (*n* = 5), and hair loss (*n* = 4). The sequential listing of interviewed participants in Table 4 illustrates the degree of saturation of symptomatic AEs, as well as the frequency counts. The saturation grid demonstrates that the sixteenth and seventeenth interviewed participants did not mention any symptomatic AEs unique to what had been mentioned by the prior fifteen participants. Some descriptions from patients about symptomatic AEs are provided:

*“My diet was terrible at first. When I first got home, you know, I could only eat very little in the way of it…it was lack of appetite. It had nothing to do with the food. It was just the lack of appetite.”*

*“I felt like I had to urinate and pee and I felt like throwing up, and I felt like, I would start sweating profusely. I said, you know, if this happens again, the nurse said and I said, well, just let me try it [Y90 treatment]. I’ll go, I promise I’ll go to bed, and I did. I went straight to bed and it just knocked me out.”*

*“I mentioned to the doctor that I was experiencing a lot of itching. Not rash, but itching, because it tells you right on the thing that you may or may not experience a rash and a whole mess of other things. But a lot of itching, and eventually that went away during the course of the year. But then, of course, then I started losing weight…and then I experienced other things like loss of appetite.”*

*“My hair just started falling out by the handful, and so that went on for quite a while. You know, I always shed tons of hair and still have tons of hair, and it was, my hair was falling out and really thin and stuff. And then after, I don’t know, maybe 5 or 6 months, it quit falling out so much.”*

**Table 4:** Saturation grid

Sex	F	M	M	M	M	M	M	M	F	M	M	M	M	M	M	M	M
Age, years	54	62	73	57	66	68	77	84	60	76	65	82	76	64	69	64	72
Treatment-related side effects; n (%)																	
Nausea; 6 (35.3%)	x			o				o	o					o		o	
Diarrhea; 2 (11.8%)	x													o			
Swelling in legs; 2 (11.8%)	x															o	
Skin breaks/cracks; 1 (5.9%)	x																
Constipation; 3 (17.6%)			x		o				o								
Vomiting; 2 (11.8%)				x		o											
Fatigue/lack of energy/tiredness; 10 (58.8%)				x		o	o		o	o	o	o	o			o	o
Pain/soreness in/around liver; 3 (17.6%)				x										o		o	
Pain/tenderness at insertion/injection site; 5 (29.4%)				x		o						o			o	o	
Blisters on feet; 1 (5.9%)				x													
Soreness around incision; 1 (5.9%)					x												
Flu-like feeling; 1 (5.9%)						x											
Lack/loss of appetite/interest in food; 6 (35.3%)						x		o		o		o			o	o	
Hair loss; 4 (23.5%)							x	o	o								o
Weakness; 1 (5.9%)							x										
Increased sweating; 1 (5.9%)								x									
Uncontrollable urination; 1 (5.9%)								x									
Urinary urgency; 1 (5.9%)								x									
Uncontrollable defecation; 1 (5.9%)								x									
Slept a lot after procedure; 1 (5.9%)								x									
Itching; 1 (5.9%)								x									
Memory issues; 2 (11.8%)								x	o								
Weight loss; 1 (5.9%)								x									
Longer to stop bleeding; 2 (11.8%)									x					o			
Pain on inside/outside of legs; 1 (5.9%)									x								
Pain under ribs near right kidney; 1 (5.9%)									x								
Chills; 1 (5.9%)										x							
Clogged left ear; 1 (5.9%)										x							
Nosebleeds; 1 (5.9%)										x							
Headaches; 2 (11.8%)											x		o				
Distended abdomen that causes pain; 1 (5.9%)														x			
Gas (feeling like needing to burp); 1 (5.9%)														x			
Unpleasant taste in mouth; 1 (5.9%)															x		

Participants are listed sequentially, from left to right, in the order in which interviews were completed

x, first mention by patient; o, subsequent mention by patient

**Fig. 2:**
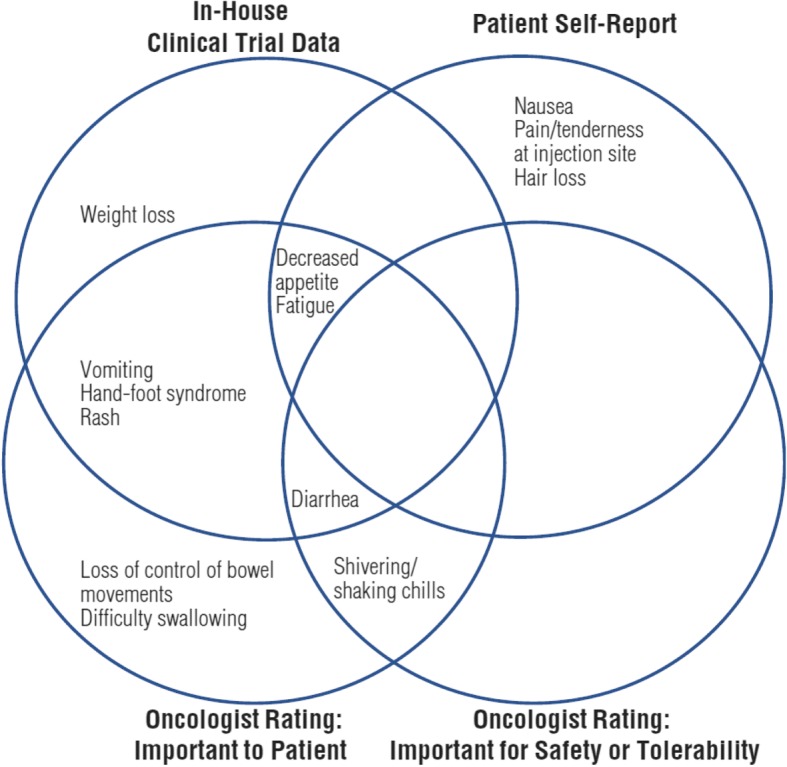
Source of information for specific symptomatic adverse events. Venn diagram showing symptomatic adverse events highly rated by clinicians and important to patients

Figure 2 depicts the overlap of the oncologist and patient feedback on thirteen symptomatic AEs. These thirteen symptomatic AEs were those with an average pooled oncologist rating score ≥7 and/or endorsed by four or more patients. Decreased appetite and fatigue were frequently reported by patients and were highly rated by oncologists as being important to patients. As stated above and shown in Fig. 2, patients frequently reported nausea, pain/tenderness at insertion/injection site, and hair loss; these symptomatic AEs were not highly rated by clinicians. Conversely, loss of control of bowel movements and difficulty swallowing were highly rated by clinicians, but mentioned by one and zero patients, respectively.

## Discussion

This study highlights the value of combining available data with clinician and patient input to identify a cancer-specific subset of PRO-CTCAE items. As seen in Fig. 2, we identified seven important symptomatic AEs based on clinical expert feedback on safety data from sponsor-conducted clinical trials. Clinical expert feedback on PRO-CTCAE items identified three additional symptomatic AEs as important. Finally, interviews with patients contributed another three symptomatic AEs. Overall, a preliminary list of 13 clinically relevant symptomatic AEs in HCC were identified using information from sponsor-conducted clinical trials, qualitative interviews with medical oncologists, and patients with HCC. Each source of information contributed at least one unique symptomatic AE. Had we not used all three sources, important AEs for measurement in HCC trials could have been overlooked. The 13 symptomatic AEs identified were hand-foot syndrome, diarrhea, fatigue, decreased appetite, rash, nausea, vomiting, weight loss, shivering/shaking chills, loss of control of bowel movements, difficulty swallowing, pain/tenderness at the injection site, and hair loss (Fig. 2).

Some of the 13 symptomatic AEs identified in this study are the same treatment-related AEs reported with nivolumab or regorafenib, systemic therapies that were recently approved for patients with advanced HCC. Diarrhea, decreased appetite, fatigue, weight decrease, and nausea were among the most frequent treatment-related AEs in patients with advanced HCC who were treated with nivolumab and were among the 13 symptomatic AEs reported in this analysis [16]. Likewise, commonly reported treatment-related AEs with regorafenib included hand-foot skin reaction, diarrhea, fatigue, nausea, weight loss, and vomiting, which are also covered in this list [15]. However, pruritus and abdominal pain were commonly reported in patients treated with nivolumab and regorafenib, respectively, but were not included among the symptomatic AEs identified in this study. Although there is some overlap in the list of symptomatic AEs identified in this study (of patients in whom a majority received locoregional therapy) with treatment-related AEs reported in patients who received new immune checkpoint or multikinase inhibitors, notable differences indicate that identification of relevant symptomatic AEs that could potentially be used as PRO-CTCAE items will likely be specific to a given class of antitumor therapy.

Because the focused symptomatic AE lists are intended for use in clinical trials that evaluate new treatments, it is important to include symptomatic AEs that cover all relevant toxicities of the treatments under study. It may be of value to target lists to different treatment types, because a list based on chemotherapies may not be completely pertinent to immunotherapies since it may not include immune-related adverse events. In our study, in spite of the small sample, we observed considerable heterogeneity of patient experiences with a variety of treatment exposures: the fact that 33 symptomatic AEs were mentioned by patients, of which 19 were endorsed by only one participant, and only one (fatigue) was mentioned by more than half, demonstrates the importance of including patients with a full range of treatment exposures. Furthermore, if patients are naïve to the experimental treatment, this could bias item selection against active comparators. The PRO-CTCAE includes an open-text field option for reporting experiences with AEs not represented in the library, such as weight loss. There also exists a process by which users of the PRO-CTCAE can partner with the National Cancer Institute to develop candidate items for use in research applications.

Another consideration is that all patients interviewed for this study were from the United States, whereas the medical oncologists were an international sample. Treatments for HCC and symptomatic AE reporting might vary based on geographic location. For example, in the GIDEON (Global investigation of therapeutic decisions in hepatocellular carcinoma and of its treatment with sorafenib) study, global variations were reported in the frequency and number of TACE treatments before sorafenib [20]. This difference in patterns of HCC treatment could affect the symptomatic AEs reported by patients. Thus, it may be important for future studies to include patients from the same countries as the clinicians enrolled in the study.

It is possible that the medical oncologists could have identified additional important symptomatic AEs. The list of AEs that they reviewed from sponsor-conducted clinical trials was generated from Grade ≥2 AEs. Had AEs of Grade 1 been presented they may have provided different feedback. However, Grade 1 AEs by definition imply ‘no intervention required’ and including them could have increased the number of non-specific items, thus potentially undermining the goal of the study, which was to develop a salient subset of PRO-CTCAE items for HCC. Interestingly, the medical oncologists did not identify some events observed in prior nivolumab and regorafenib trials in HCC. This suggests one cannot assume clinicians will identify all relevant events for drugs under study/yet to be approved. 

Finally, socioeconomic status (SES) is a barrier that could limit oncology patient input [21, 22]. A growing body of evidence indicates that oncology patients of low SES have worse outcomes and a survival disadvantage compared with patients of higher SES. SES may be particularly relevant for a condition such as HCC [23, 24] because chronic viral hepatitis (a major risk factor for HCC) is correlated with substance abuse behaviors, which are in turn associated with poor education and unemployment [25, 26]. Thus, it is important for future studies to investigate how applicable the 13-item subset identified in this study is to patients with HCC who are of low SES.

Combining clinician and patient input enabled us to identify a more comprehensive set of AEs, including weight loss, an event not currently represented in the PRO-CTCAE library. Some of the symptomatic AEs such as fatigue/tiredness and lack of appetite were reported by both medical oncologists and patients. However, symptomatic AEs such as skin toxicities and diarrhea were reported commonly by medical oncologists but not by patients; patients frequently reported nausea, pain/tenderness at insertion/injection site, and hair loss in addition to fatigue/tiredness and lack of appetite. These discrepancies noted between patient and clinician input in this study highlight the importance of combining their perspectives and are in agreement with previous work that has also reported differences in clinician and patient reports of symptomatic AEs [6–8].

Evidence for the validity of this preliminary 13-item set of symptomatic Es should be collected in future HCC clinical trials. The methodology applied herein suggests that a relatively small subset of PRO-CTCAE items can be identified for application within specific tumors, using information from clinical trials and a limited number of physician and patient interviews. Inclusion of the patient perspective, rather than reliance on clinician reporting alone or previous information from sponsor clinical trials, helps capture subjective elements of safety information that may have both clinical and scientific value [1].

## Conclusion

In summary, this study identified clinically relevant symptomatic AEs from both medical oncologists and patients with HCC that can be used in evaluating treatment tolerability in HCC clinical trials. Through the methodology applied herein, we were able to reduce the 78 symptomatic AEs represented in the PRO-CTCAE library to a set of 13 events warranting further exploration. These results support incorporating both patient and clinician perspectives in identifying PRO-CTCAE items for use in cancer clinical trials.

## Abbreviations

AE: Adverse event
CTCAE: Common Terminology Criteria for Adverse Events
HCC: Hepatocellular carcinoma
PRO: Patient-reported outcome
PRO-CTCAE: Patient-Reported Outcomes version of the Common Terminology Criteria for Adverse Events
SES: Socioeconomic status
TACE: Transarterial chemoembolization
